# Optimizing the utility of NIMR-MDB vis a vis ICMR COVID-19 data portal in containment of malaria

**DOI:** 10.1016/j.lansea.2022.100044

**Published:** 2022-07-18

**Authors:** Gaurav Kumar, Hari Shankar

**Affiliations:** a ICMR-National Institute of Malaria Research, Sector-8, Dwarka, Delhi, 110077, India; b Indian Council of Medical Research, V. Ramalingaswami Bhawan, Ansari Nagar, New Delhi, 110029, India

Undoubtedly, National Institute of Malaria Research-Malaria Dashboard (NIMR-MDB) by Yadav and colleagues (2022) is a useful resource for researchers.^1^ However, real time reporting of data will be of better utility to policymakers for malaria containment in India. Yadav et al. created the MDB using the data available with the National Centre for Vector Borne Diseases Control (NCVBDC); however, we advocate integrating the malaria data in real-time manner. Analysis of this real time data would help in foreseeing the up-surged malaria cases in a region and devising its control strategies in advance.

There could be two plausible approaches to integrate the data, first is the use of the Indian Council of Medical Research (ICMR) COVID-19 data portal. ICMR COVID-19 data portal was established for tracking of COVID-19 cases in real-time at primary health centre (PHC) level. Therefore, incorporation of real time malaria data in this portal could be done without putting additional efforts in terms of cost/man-power/resources/infrastructures etc. Another approach is the use of mobile-based applications at the PHC level for online reporting. Fever Tracker is one such app which has proven instant digitization of malaria data for further handling.^2^

The authors were engaged in the COVID-19 case reporting on the ICMR and Uttar Pradesh (UP) COVID-19 data portal.^3^ UP COVID-19 portal was initially standalone used for COVID-19 tracking in Uttar Pradesh state. Later on, UP COVID-19 portal was integrated with ICMR COVID-19 data portal. Similarly, the utility of NIMR-MDB to estimate the burden and trend of malaria can be enhanced by integrating it with ICMR COVID-19 data portal.

## Contributors

H.S.: Conceptualization, Writing of article: G.K. and H.S.

## Declaration of interests

None.
